# Respiratory Distress Syndrome: An Important Risk Factor for Intraventricular Hemorrhage in Preterm Neonates

**DOI:** 10.7759/cureus.116165

**Published:** 2026-09-13

**Authors:** Melika Dzeko, Sabina Terzic, Sadzida Orucevic

**Affiliations:** 1 General Medicine, Public Institution Health Center of Sarajevo Canton, Sarajevo, BIH; 2 Pediatrics, Clinical Center University of Sarajevo, Sarajevo, BIH

**Keywords:** intraventricular hemorrhage, mechanical ventilation, preterm infant, respiratory distress syndrome, risk factors

## Abstract

Introduction: Intraventricular hemorrhage (IVH) represents the most common pathology of the nervous system in neonates born before the 32nd week of gestation. Multiple factors present a risk for the development of IVH. It is important to know these factors for potential prevention and adequate treatment.

Methodology: A retrospective clinical study was conducted at the Neonatal Intensive Care Unit of the Pediatric Clinic, Sarajevo University Clinical Center. Data were collected for 157 neonates in the period from 1 July 2023 to 31 December 2024.

Results: Of the 157 premature neonates born at <32 weeks of gestation, 53 (33.8%) had IVH. We found that newborns with IVH had lower gestational age, birth weight, and Apgar scores, especially at the fifth minute. Contrary to many studies, the use of antenatal corticosteroids showed no protective effect. Newborns with respiratory distress syndrome (RDS) had a two-times higher relative risk for the development of IVH. Analysis showed a significant correlation between the duration of mechanical ventilation (MV) of more than three days and the incidence of IVH (24.1% vs. 54.5%; χ²=11.56; p<0.001), as well as between inotropic therapy and IVH (35.8% vs. 8.7%; χ²=17.72; p<0.001). In the group of children with a lethal outcome, 83.3% had IVH, compared with 16.7% who died without a diagnosis of IVH.

Conclusion: RDS, lower gestational age, birth weight and Apgar score, MV longer than three days, and inotropic therapy were found to be important risk factors for the development of IVH in neonates born before the 32nd week of gestation.

## Introduction

Due to disrupted development, prematurely born children have numerous complications and a higher risk of unfavorable outcomes. The most common complications in these babies include respiratory distress syndrome (RDS), patent ductus arteriosus (PDA), sepsis, necrotizing enterocolitis (NEC), intraventricular hemorrhage (IVH), retinopathy of prematurity (ROP), bronchopulmonary dysplasia (BPD), cerebral palsy, and intellectual disability [1].

The Vermont Oxford Network (VON) defines neonatal RDS using clinical and radiological criteria: oxygen needs (PaO_2_ <50 mmHg or O_2_ saturation >85%) in the first 24 hours, central cyanosis, plus characteristic chest X-ray findings such as ground-glass opacities, often due to surfactant deficiency in premature infants, requiring oxygen and sometimes ventilation.

PDA is a common congenital heart defect in which a normal fetal blood vessel, the ductus arteriosus, fails to close shortly after birth, allowing oxygenated blood to flow from the aorta back to the lungs [2].

The American Academy of Pediatrics recognizes BPD as a chronic lung disease of prematurity, essentially defined by the need for supplemental oxygen for at least 28 days (or longer) and often requiring mechanical ventilation (MV). It stems from lung damage in premature infants due to early oxygen and ventilator use, leading to ongoing breathing problems, with evolving definitions focusing on severity at 36 weeks postmenstrual age.

IVH is the most common neurological complication in very preterm infants and is detected by cranial ultrasonography in premature newborns born before the 32nd week of gestation [3]. The incidence of IVH in children is higher at lower gestational ages: 41% at 28 weeks and 28% at 32 weeks [4]. IVH most often occurs as venous bleeding from fragile blood vessels that vascularize the germinal matrix. The germinal matrix is a site of neuronal and glial proliferation that is complete approximately by the end of the 32nd week of gestation. The site is very rich in mitochondria and therefore sensitive to ischemia [5]. This area has high metabolic activity, but the blood vessels are fragile, underdeveloped, and very often proliferate, which is why this location is susceptible to bleeding [6]. IVH occurs nearly exclusively in the first week of life, and newborns become relatively immune to hemorrhage after that period, irrespective of gestational age. This could be due to an increase in oxygen concentration in the blood and tissue, suppressing endothelial growth factor and angiopoietin-2, which stops angiogenesis and enhances the maturation of vessels, making them less susceptible to rupture [7]. According to some studies, the global incidence of IVH is 36.2%, of which grades III and IV are present in 7.1% [8]. Risk factors for IVH include immaturity of the blood vessels, fluctuation of cerebral blood flow, lack of autoregulation, fluctuation of venous blood pressure, variations in venous anatomy, as well as genetic factors. These factors, both individually and in combination, contribute to the development of hemorrhage [7]. Other factors with a significant role in IVH are low birth weight, prolonged MV, low APGAR score in the fifth minute, hypoxia-induced damage, hypothermic ischemia, pneumothorax, thrombocytopenia, abnormal acid-base homeostasis, antenatal maternal hemorrhage, maternal infection, sepsis, hypotension, hypoxia, hypercapnia, seizures, PDA, infections, and RDS [9].

The most commonly used classification systems for IVH are those by Papile and Volpe [7]. In both systems, we differentiate four grades. The classification system by Volpe includes: Grade I - germinal matrix hemorrhage, with little to no bleeding in the ventricles, involving <10% of the ventricular area on the parasagittal view; Grade II - IVH occupying 10% to 50% of the ventricular area in the parasagittal view; Grade III - IVH occupying greater than 50% of the ventricle, with or without ventricular echodensities; and Grade IV - periventricular hemorrhagic infarction. The classification system by Papile includes: Grade I - hemorrhage limited to the germinal matrix; Grade II - blood noted within the ventricular system but not distending it; Grade III - blood in the ventricles with distension of the ventricles; and Grade IV - IVH with parenchymal extension [10].

Up to 50% of IVH cases involving the germinal matrix are asymptomatic. Generally, lower-grade hemorrhages are often found during routine examinations [11]. In children with severe IVH and periventricular hemorrhage, the first symptoms could be hyperbilirubinemia (without other explanation), changes in the level of consciousness, cardiopulmonary symptoms, lower hematocrit, acidosis, hypoglycemia/hyperglycemia, tense fontanelle, hypotonia, lethargy, and seizures. Inappropriate release of antidiuretic hormone can also be found in these babies [12]. Severe bleeding can lead to deterioration with stupor, hypotonia, generalized tonic seizures, and severe cardiocirculatory instability [8]. The most commonly used diagnostic tool is transcranial ultrasound [13]. MRI of the brain is useful for the identification of cerebellar hemorrhages, white matter injury, and brain anomalies. CT is a good imaging method for calcifications, bleeding, and fractures [14].

There is no specific treatment for IVH. It is important to constantly monitor the progression of the bleeding and the width of the ventricles in order to prevent further neurological deficits. Complications of IVH can be short-term and long-term. Short-term complications are expansion of the hemorrhage and transient ventricular enlargement, while long-term complications are posthemorrhagic hydrocephalus and neurodevelopmental sequelae, such as motor and cognitive developmental delays, potential seizures, and cerebral palsy [5]. In higher grades of IVH (>III), expected complications are periventricular hemorrhagic infarction, posthemorrhagic ventricular dilation, cerebellar hemorrhagic injury, and periventricular leukomalacia, which can affect neonatal morbidity, mortality, and long-term neurodevelopmental outcomes [15]. Neonates with grade I and II IVH have a lower risk of developing sequelae but still need careful neurodevelopmental monitoring. One of the basic preventive measures that is highly recommended for all mothers at risk of premature birth is antenatal corticosteroids. Other measures include delayed umbilical cord clamping, maintaining a euthermic state, decreasing the rate of MV, and subtle modes of surfactant application (less invasive surfactant administration (LISA) and minimally invasive surfactant therapy (MIST)) [3]. Measures for maintaining stability and avoiding fluctuations in cerebral blood flow and blood pressure are also important, including adequate care or closure of PDA, keeping the child in a supine position with the head in the central line, and aspiration of foreign material via the endotracheal tube only when necessary.

This study aimed to identify the most important risk factors that contribute to the development of IVH, especially focusing on MV, RDS, and hemodynamic instability (presence of PDA and inotrope use), as well as examining some preventive measures, such as antenatal corticosteroid administration, that could lower the risk of IVH development or some of its severe consequences.

## Materials and methods

This is a single-center retrospective cohort study conducted at the Neonatal Intensive Care Unit of the Pediatric Clinic of the Sarajevo University Clinical Center. The NICU annually admits 450-500 patients from 23+0 gestational weeks. This is the highest-level NICU in our country, covering a region of one million inhabitants. Data were collected from 230 electronic medical records, which finally included 157 neonates during the period from 1 July 2023 to 31 December 2024. Data were collected by a final-year medical student under the supervision of an experienced neonatologist.

We included preterm infants born between 23+0 and 31+6 GW who had cranial ultrasound performed. Exclusion criteria were major congenital anomalies and syndromes. Analysis was conducted exclusively on complete datasets, with all observations containing missing values being excluded. The following variables were collected: gender, gestational age, birth weight, first- and fifth-minute Apgar scores, antenatal corticosteroid administration, RDS, use and duration of MV, inotrope administration, sepsis, NEC, brain ultrasound results (presence and grade of IVH according to the Papile grading system), patient outcome, PDA, BPD, and ROP. Brain ultrasound was performed by experienced, licensed neonatologists. Ultrasound was performed for each patient once during the first three days of life and on the seventh and 28th days of life in patients who had not had a fatal outcome until then. Ventilation protocols in the NICU setting follow the European Consensus Guidelines on the Management of Respiratory Distress Syndrome.

Variables were retrospectively confirmed for this study using the available data in the medical records. The use of surfactant treatment and prolonged oxygen supplementation, with confirmation by lung X-ray, were criteria for the diagnosis of RDS. PDA was diagnosed based on echocardiography, and treatment was administered according to the advice of cardiologists. Sepsis was diagnosed only if culture-proven. Hemodynamic instability was confirmed based on noninvasive monitoring and echocardiography.

Statistical analysis was conducted using R statistical software (the latest version of RStudio available at the time of the analysis). For univariate analysis, descriptive statistics were used (mean, standard deviation, median, interquartile range, minimum, and maximum values), depending on the distribution of the data.

For comparison of continuous variables between two groups, the independent t-test for parametric data and the Mann-Whitney U test for nonparametric data were used. Categorical variables were analyzed using the chi-square test and Fisher's exact test, depending on the expected frequencies. To estimate correlations between variables, the Spearman rank correlation coefficient and Kendall's tau-b coefficient were used. IVH was analyzed using regression models appropriate to the structure of the outcome variables. For the primary analysis, IVH was treated as a binary outcome (presence vs. absence), and associations were assessed using multivariable binary logistic regression. Gestational age was selected a priori for inclusion in the primary model as a marker of biological maturity.

For the secondary analysis, IVH severity was categorized as no IVH, grade I-II IVH, and grade III-IV IVH, and analyzed using multinomial logistic regression, with no IVH as the reference category. Birth weight was included in this model to evaluate its association with increasing IVH severity.

Gestational age and birth weight were not included simultaneously in the same model because of collinearity. Other covariates were selected a priori based on clinical relevance and temporal precedence and included sex, multiple pregnancy, mode of delivery (cesarean vs. vaginal), antenatal corticosteroid exposure, and mode of conception (in vitro fertilisation (IVF) vs. non-IVF). Results are presented as adjusted odds ratios (aORs) with 95% confidence intervals (CIs). No adjustment for multiple comparisons was applied, as the analyses focused on predefined primary and secondary outcomes. The level of statistical significance was set at p<0.05.

Although this was a retrospective study, approval was obtained from the Ethics Committee of the Clinical Center (approval number 51-30-5-8994/25).

## Results

During the period from 1 July 2023 to 31 December 2024, 157 neonates fulfilled the inclusion criteria. Of the 157 neonates, 53 (33.8%) had IVH. There were 66.1% of patients with lower-grade hemorrhages (>III).

Neonates with IVH had a significantly lower gestational age compared with those without IVH (28.0±2.5 vs. 30.1±2.0 weeks; p<0.001). Similarly, birth weight was significantly lower in the IVH group (1181.5 g) than in the non-IVH group (1527.6 g), with the Mann-Whitney U test confirming a statistically significant difference (U=1451.0; Z=-4.723; p<0.001) (Table 1).

**Table 1:** Characteristics of patients according to IVH/non-IVH group Statistical significance was set at p<0.05. t, t-test; U, Mann-Whitney U statistic; Z, standardized test statistic for the Mann-Whitney U test; χ², Chi-square test; Fisher's, Fisher's exact test;  RDS, respiratory distress syndrome; MV, mechanical ventilation; PDA, patent ductus arteriosus; IVH, intraventricular hemorrhage

Characteristics	IVH	Non-IVH	Test	P-value
Gestational age (weeks) characteristics	28.0±2.5	30.1±2.0	t=5.74	<0.001
Birth weight (mean, grams)	1181.5	1527.6	U=1451.0, Z=-4.723	<0.001
Apgar 1^st^ min	5.8	6.8	t=2.725	0.004
Apgar 5^th^ min	7.8	7	t=2.710	0.006
RDS (%)	81.1%	60.6%	χ²(1)=6.764	0.009
MV<3 days (%)	24.1%	75.9%	χ²(1)=11.56	<0.001
MV<3 days (%)	54.5%	45.5%		
Inotrope (%)	35.8%	8.7%	χ²(1)=17.72	<0.001
PDA (%)	85.7%	14.3%	Fisher's	<0.001
Died (%)	83.3%	16.7%	χ²(1)=31.14	<0.001

Respiratory morbidity was strongly associated with IVH. RDS was present in 81.1% of infants with IVH compared with 60.6% of those without IVH, and this difference was statistically significant (χ²=6.764; p=0.009). Infants with RDS had approximately twice the risk of developing IVH compared with those without RDS. In addition, the duration of MV was significantly associated with the occurrence of IVH. Among infants ventilated for up to three days, IVH was observed in 24.1% of cases, whereas in those requiring more than three days of MV, IVH occurred in 54.5% of cases (χ²=11.56; p<0.001) (Table 1).

Hemodynamic instability was also significantly associated with IVH. Inotropic support was required in 35.8% of infants with IVH, compared with only 8.7% of infants without IVH. This difference was highly significant (χ²=17.72; p<0.001), indicating that the need for inotropic therapy was strongly associated with an increased probability of IVH. Furthermore, PDA showed a strong association with the occurrence of IVH. Among neonates with PDA, IVH was present in 85.7% of cases, compared with 14.3% among those without PDA (Fisher's exact test, p<0.001) (Table 1).

A strong association was observed between IVH and neonatal outcome. IVH was present in 83.3% of infants who died, compared with 16.7% among survivors, and this difference was highly statistically significant (χ²=31.14; p<0.001). Moreover, analysis of IVH severity demonstrated a moderate negative correlation between IVH grade and outcome, indicating that infants with lower IVH grades had better outcomes (Kendall's tau-b=-0.488; p<0.001) (Table 1).

No significant association was found between antenatal corticosteroid exposure and IVH occurrence. IVH was present in 34.8% of neonates who did not receive antenatal corticosteroids and in 33.0% of those who did, with no statistically significant difference between the groups (χ²=0.061; p=0.806).

To further evaluate independent predictors of IVH and its severity, multivariable regression analyses were performed, including binary logistic regression for IVH occurrence and multinomial logistic regression for IVH severity (Table 2).

**Table 2:** Binary and multinomial logistic regression for IVH The results of the multivariable regression analyses for IVH are presented. In the binary logistic regression model, lower gestational age was independently associated with higher odds of IVH, whereas sex, multiple pregnancy, mode of delivery, antenatal corticosteroid exposure, and mode of conception were not significantly associated with IVH after adjustment. In the multinomial logistic regression model assessing IVH severity, lower birth weight was independently associated with both mild (grade I-II) and severe (grade III-IV) IVH, with a stronger effect observed for severe IVH. Mode of conception by IVF was not associated with mild IVH but was strongly associated with severe IVH compared with no IVH, while no other covariates showed statistically significant independent associations with IVH severity. ^*^Statistical significance was set at p<0.05. aOR, adjusted odds ratios; CI, confidence intervals; IVH, intraventricular hemorrhage; IVF, in vitro fertilization

Binary logistic regression for IVH (yes/no) - (primary outcome; model including gestational age)						
Variable		aOR		95% CI	P-value	
Gestational age (per 1 week)		0.64		0.53-0.76	<0.001	
Sex		1.34		0.59-3.02	0.48	
Multiple pregnancy		0.47		0.17-1.29	0.14	
Caesarean delivery		0.64		0.28-1.45	0.28	
Antenatal corticosteroids		0.81		0.36-1.79	0.60	
Mode of conception-IVF		1.92		0.46-8.06	0.37	
Multinomial logistic regression for IVH severity (secondary outcome; reference category: no IVH; model including birth weight)						
IVH grade III-IV vs. no IVH (mild grade)						
Variable	aOR		95% CI			P-value
Birth weight (per 1 g)	998		0.997-0.999			^*^<0.001
Sex	1.81		0.73-4.51			0.20
Multiple pregnancy	0.55		0.17-1.75			0.31
Caesarean delivery	0.44		0.17-1.09			0.07
Antenatal corticosteroids	0.88		0.36-2.14			0.78
Mode of conception-IVF	1.92		0.46-8.06			0.37
IVH grade III-IV vs no IVH (severe grade)						
Variable	aOR		95% CI			P-value
Birth weight (per 1 g)	994		0.991-0.997			<0.001
Sex	3.40		0.83-13.90			0.09
Multiple pregnancy	0.12		0.01-1.22			0.07
Caesarean delivery	0.41		0.10-1.76			0.23
Antenatal corticosteroids	0.50		0.12-2.05			0.33
Mode of conception-IVF	22.6		2.63-195.3			0.004

## Discussion

In the study, we investigated the occurrence of IVH in premature neonates born before 32 weeks of gestation. Out of a total of 157 patients, 53 (33.8%) had IVH. In the study by Behnaz Baisiri et al. [9], 21.4% of patients had IVH, while in the study by Piscopo et al. [3], 20.02%, and in the study by Nagy et al. [4], 27%.

The week of gestation proved to be a significant risk factor for the development of IVH. The difference between the groups of neonates that had and did not have IVH was 2.1 weeks, which was statistically significant and indicates that prematurity and general underdevelopment of the newborns have an important role in IVH development. These same conclusions were shown in the work of Rhee CJ et al. [16] and Eren Özek and Kersin SG [12].

We found a significant difference in birth weight between neonates with and without IVH and showed that lower birth weight was an important risk factor for the development of IVH. The same was found by Praveen Kumar and Mounika Polavarapu [17], as well as Ying Zhao et al. [18] in their studies.

Our research found a negative correlation between the Apgar score and the development of IVH, with lower Apgar scores, especially at the fifth minute, being associated with a higher incidence of IVH. Many other authors, including Starr R. et al. [19] and Egesa WI et al. [20], presented similar results. The most plausible explanation is that hypoxic-ischemic mechanisms, hemodynamic instability, and the necessity for intensive interventions during the immediate postnatal period contribute to an increased vulnerability to hemorrhage development.

Contrary to the findings of Lejiser LM and de Vries LS [21] and Kebede et al. [22], our study showed no significant association between antenatal corticosteroid administration and the development of IVH. This may be explained by the relatively small number of mothers who received antenatal corticosteroids and the single-center design of the study.

RDS and the length of MV were proven to be key risk factors for the development of IVH in our research. In the study, neonates with RDS had a two-times higher risk of developing IVH. There was also a statistically significant difference in IVH development between neonates who were on ventilation support for less than three days and neonates who required longer support. As MV increases intrathoracic pressure, it prevents blood return to the heart from the brain and can potentially cause IVH. Interestingly, Yong Wang et al. [23], as well as Tain Wu et al. [24], showed that RDS does not have much influence on hemorrhage development. Other studies by Piscopo et al. [3] and Ying Zhao et al. [18] are consistent with our results that MV is an important risk factor.

In our study, hemodynamic variables proved to be statistically significant factors for IVH development. Inotrope use significantly increased the probability of IVH development. The same observation was shown by Stathopoulou et al. [25], as well as by Alghadam et al. [26]. When it comes to PDA, our study confirms the significant connection between PDA presence and IVH development. PDA causes hemodynamic instability and impaired cerebral blood flow, which result in IVH. The same observations come from Khanafer-Larocque I et al. [2] and Al-Mouqdad et al. [27], but not from Wu et al. [24].

Consistent with previous studies, we found a correlation between high-grade IVH and lethal outcome. Kan A. et al. [28] came to the same conclusion, as did Rees et al. [29] and many others.

Limitations of our research were incomplete medical records, which decreased the number of patients included in the single-center study. Another limitation was a relatively small sample in subgroups and, therefore, results that did not correlate with expected outcomes. For example, the small sample of women who received antenatal corticosteroids showed that corticosteroids did not have a protective effect on the development of IVH. The most important limitations of the study are its retrospective and single-center design, so the generalizability of our findings to other institutions, populations with different demographic characteristics, and clinical practices may be limited. Future studies should focus on a prospective and multicenter approach.

## Conclusions

RDS, lower gestational age, birth weight, and Apgar score, as well as the need for MV longer than three days, are associated with an increased risk of developing IVH in neonates born before the 32nd week of gestation. These babies have a significantly increased risk of mortality and unfavorable neurological outcomes, so it is of great importance to identify and possibly prevent risk factors. Lower birth weight and gestational age cannot always be prevented. A lower Apgar score, especially in the fifth minute of life, is a significant predictive factor for IVH development, and therefore, we must make efforts to optimize neonatal resuscitation practices. As RDS and MV are strongly associated with IVH development, it is necessary, whenever possible, to apply noninvasive ventilation modalities and gentle surfactant application (LISA, MIST). Maintaining hemodynamic stability may improve the chances of a lower incidence of IVH.
